# The immunotherapy era in ovarian clear cell carcinoma: current evidence and future perspective

**DOI:** 10.3389/fimmu.2025.1661048

**Published:** 2025-11-26

**Authors:** Anna Passarelli, Sabrina Chiara Cecere, Jole Ventriglia, Carmela Pisano, Rosella De Cecio, Sabrina Rossetti, Rosa Tambaro, Marilena Di Napoli, Lorenzo Lobianco, Gabriele Calvanese, Maria Rosaria Lamia, Erica Perri, Maria Sara Peluso, Emilia Scarpa, Salvatore Stilo, Francesco Fiore, Sergio Venanzio Setola, Daniela Califano, Sandro Pignata

**Affiliations:** 1 Department of Urology and Gynecology, Istituto Nazionale Tumori Istituto di Ricovero e Cura a Carattere Scientifico (IRCCS) Fondazione G. Pascale, Naples, Italy; 2 Division of Anatomic Pathology and Cytopathology, Istituto Nazionale Tumori Istituto di Ricovero e Cura a Carattere Scientifico (IRCCS) Fondazione G. Pascale, Naples, Italy; 3 Medical Oncology, Department of Precision Medicine, University of Campania Luigi Vanvitelli, Naples, Italy; 4 Department of Oncology and Hemato-Oncology, Università degli Studi di Milano, Milan, Italy; 5 Department of Clinical Medicine and Surgery, University Federico II, Naples, Italy; 6 Interventional Radiology Unit, Istituto Nazionale Tumori Istituto di Ricovero e Cura a Carattere Scientifico (IRCCS) Fondazione G. Pascale, Naples, Italy; 7 Radiology Unit, Istituto Nazionale Tumori Istituto di Ricovero e Cura a Carattere Scientifico (IRCCS) Fondazione G. Pascale, Naples, Italy; 8 Microenvironment Molecular Targets Unit, Istituto Nazionale Tumori Istituto di Ricovero e Cura a Carattere Scientifico (IRCCS) Fondazione G. Pascale, Naples, Italy

**Keywords:** ovarian clear cell carcinoma, *ARID1A* mutations, tumor microenvironment, immune checkpoint inhibitors, combination immunotherapy

## Abstract

Ovarian clear cell carcinoma (OCCC) is a rare, aggressive epithelial ovarian cancer subtype, accounting for approximately 10% of cases and associated with a poor prognosis due to chemoresistance and unique tumor biology. OCCC is frequently linked to endometriosis and characterized by mutations in *ARID1A* and *PIK3CA*, hyperactivation of the PI3K/Akt/mTOR pathway, and overexpression of VEGF, HIF-1α, and IL-6. These features drive tumor proliferation, angiogenesis, immune evasion, and resistance to platinum-based chemotherapy. The tumor microenvironment of OCCC is highly immunosuppressive, with infiltration of regulatory T cells, tumor-associated macrophages, and upregulation of immune checkpoint molecules, such as PD-1, PD-L1, and LAG-3. These characteristics suggest that the PD-1/PD-L1 pathway plays a critical role in tumor immune evasion and could be an attractive target for therapeutic intervention. Despite the typical composition of the immunosuppressive tumor microenvironment in ovarian cancer, until now overall the results of trials testing immune checkpoint inhibitors so far have been disappointing. It is interesting to note instead that several subgroup analyses reported exceptional OCCC sensitivity to ICIs. Indeed, current and preliminary trials exploring ICIs, anti-angiogenic agents, and combinatorial therapies in OCCC show promising outcomes. Strategies targeting multiple pathways, including VEGF, IL-6, HIF-1α, and HDAC6, alongside ICIs, are under investigation to overcome resistance mechanisms. Additionally, IL-10 inhibition or ferroptosis pathway activation offers novel therapeutic potential. Personalized, biomarker-driven approaches, targeting *ARID1A* and *PIK3CA* mutations or combining immune and anti-angiogenic agents, are gaining traction in OCCC management. This review highlights OCCC molecular underpinnings and therapeutic challenges, emphasizing the need for innovative, multi-targeted strategies. Advances in understanding genetic-immunological interplay in OCCC may enable more effective and durable treatments and improved patient outcomes.

## 1 Introduction

Epithelial ovarian carcinoma (EOC) is the deadliest gynecological malignancy and among the leading contributors to cancer-related mortality in women worldwide (1). Up to 90% of ovarian neoplasms are classified as EOC, further divided into the following subtypes based on histopathology: high-grade serous carcinomas, the most prevalent, comprising about 70% of cases; low-grade serous carcinomas, accounting for less than 5%; endometrioid carcinomas (EC), representing 10%; clear cell carcinomas (CCC), also accounting for 10%; and mucinous carcinomas, comprising 3% (2). Ovarian clear cell carcinoma (OCCC) is thought to arise from the malignant transformation of ectopic endometrial tissue located on the ovary. This is supported by the presence of endometriosis in 50–70% of OCCC cases (3), linked to a 2.3-fold increased risk of OCCC development (4–6). As a result, OCCC, along with EC, is classified as an endometriosis-associated ovarian cancer (7–10). OCCC represents a distinct pathological subtype, typically presenting as a large, unilateral pelvic mass that, on histopathological examination, displays a combination of papillary, tubulocystic, and solid growth patterns, along with clear and eosinophilic cells and stromal hyalinization (11). Notably, the presence of intracellular glycogen deposits is a highly distinctive diagnostic feature (12). This subtype exhibits poor responsiveness to platinum-based chemotherapy and is associated with worse outcomes than other EOC subtypes (10, 13). Furthermore, cancer-associated thromboembolism, a vascular thromboembolic complication, occurs more frequently in OCCC than in other histologic EOC subtypes, contributing to an unfavorable prognosis (14–17). When diagnosed at an advanced stage, OCCC is associated with shorter progression-free survival (PFS) and overall survival (OS) and poor chemotherapy response (18). Among patients diagnosed at FIGO stages I–II, OS rates are 80–89%, and PFS ranges from 56% to 88%. In contrast, for FIGO stages III–IV, OS drops to 52%, and PFS declines to 25% (19–22). Interestingly, OCCC is characterized by a typical tumor microenvironment (TME) with a strong immunosuppressive milieu. These characteristics suggest that the PD-1/PD-L1 axis is a key mechanism in tumor immune evasion and represents a potentially valuable target for therapeutic intervention. Although clinical trials evaluating immune checkpoint inhibitors (ICIs) in advanced ovarian cancer have so far produced mostly disappointing results, several subgroup analyses have identified a marked sensitivity of the clear cell ovarian carcinoma (OCCC) histotype to ICIs.

This review further clarifies the unique molecular signature and TME features of OCCC, focusing primarily on the promising role played by the use of immunotherapy.

## 2 Methods

Papers to be considered were identified by conducting PubMed searches, using different combinations of pertinent keywords “ovarian clear cell carcinoma,” “OCCC,” “immune checkpoint inhibitors,” “PD-1,” “PD-L1,” “LAG-3,” “ARID1A,” “PIK3CA”, “VEGF,” “tumor microenvironment,” “immune evasion,” “immunosuppression,” “HIF-1α,” “IL-6,” “angiogenesis,” “ferroptosis,” “HDAC6,” “clinical trials,” and “immunotherapy”. Boolean operators (AND, OR) were used to refine the search. Clinical trial data were retrieved from ClinicalTrials.gov using the keyword “ovarian clear cell carcinoma” and filtered for interventional studies involving immunotherapeutic agents or combination therapies. Articles were selected based on their relevance to the pathogenesis, immune modulation, and emerging therapeutic approaches in OCCC.

## 3 Results

### 3.1 OCCC molecular signature

The most frequent genetic alterations observed in OCCC include mutations in the ARID1A, KRAS, PTEN, and PIK3CA genes (23). Additional oncogenic changes affecting the mitogen-activated protein kinase (MAPK) pathway have been reported, such as mutations in PPP2R1A, mutations and amplifications of ERBB2, and amplification of the MET proto-oncogene, which encodes the hepatocyte growth factor receptor (HGFR) (24). Recently, mutations in MUC4, MAGEE1, and ARID3A have been identified at notable frequencies, with MAGEE1 mutations correlating with poorer prognosis (25). Familial inheritance is rare in OCCC, which typically expresses wild-type p53 and exhibits a very low frequency of BRCA1 and BRCA2 mutations (26), while TERT promoter mutations occur more commonly (27).

OCCC is characterized by high expression levels of napsin A, hypoxia-inducible factor 1-alpha (HIF-1α) (28), glypican-3 (29), hepatocyte nuclear factor 1-beta (HNF-1β) (30), interleukin 6 (IL-6) (31), and MET (32), whereas estrogen receptor expression is generally absent (33, 34). Although cancer antigen 125 (CA125) levels are usually low in OCCC, elevated CA125 correlates with worse prognosis (35).

ARID1A, encoding the chromatin remodeler BAF250, is involved in transcriptional regulation, DNA synthesis, and cellular proliferation and differentiation. Mutations in ARID1A occur in approximately 50% of OCCC cases and represent an early event in tumorigenesis (36). ARID1A loss impairs interferon signaling, facilitating immune evasion (37), and is associated with increased expression of HDAC6, which promotes tumor cell invasion, migration, and poorer overall survival (38–40). The co-occurrence of ARID1A and PIK3CA mutations leads to hyperactivation of the phosphoinositide 3-kinase (PI3K) pathway via p110α, stimulating the PI3K/Akt/mammalian target of rapamycin (mTOR) cascade and enhancing cellular proliferation (33, 41–44).

Recent evidence implicates the PI3K/Akt/mTOR and extracellular signal-regulated kinase 1/2 (ERK1/2) pathways in OCCC chemoresistance. These pathways regulate progranulin (PGRN) overexpression (45) and induce HIF-1α expression, which promotes metabolic adaptations including increased glycogen accumulation in PIK3CA-mutated OCCC cells and contributes to chemoresistance (46). Furthermore, Akt/mTOR signaling upregulates vascular endothelial growth factor (VEGF), driving tumor angiogenesis critical for growth, invasion, and metastasis (47). The PI3K/Akt pathway also mediates resistance to ferroptosis, an iron-dependent oxidative stress-induced form of cell death, which is regulated by the Hippo signaling pathway (48). Notably, reduced Hippo pathway activity, particularly low nuclear expression of yes-associated protein 1 (YAP1) correlates with poor prognosis and resistance to ferroptosis inducer erastin. Suppression of zinc finger DHHC-type palmitoyltransferase 7 (ZDHHC7) activates YAP1, sensitizing OCCC cells to ferroptosis, suggesting that targeting ZDHHC7 to enhance YAP1-mediated ferroptosis represents a promising therapeutic approach (49).

Decreased PTEN expression or inactivation, known to promote gastric cancer via Hippo and PI3K/Akt pathway activation (50), may similarly contribute to OCCC pathogenesis. In OCCC, low PTEN levels lead to hyperactivation of the PI3K/Akt pathway and, when combined with ARID1A mutations, promote programmed death-ligand 1 (PD-L1) expression (51).

Combined ARID1A and PIK3CA mutations also elevate IL-6 expression, a known ARID1A-regulated gene (52). PIK3CA mutations drive sustained IL-6 production through PI3K/Akt hyperactivation in the context of ARID1A loss (36). IL-6 maintains JAK/STAT3 signaling, amplifying its own expression and promoting resistance to oxidative stress, tumor invasion, and chemoresistance (53–56). IL-6 additionally induces nuclear translocation and transcriptional activation of HIF-1α via STAT3, enhancing cisplatin resistance in ovarian cancer cells (57). HIF-1α, highly expressed in OCCC, contributes to glycogen accumulation, metabolic adaptation, and activation of IL-6 signaling (28, 46, 58). Through STAT3 activation, IL-6 induces VEGF expression, promoting angiogenesis and vascular permeability. Correspondingly, ARID1A mutations correlate with elevated VEGF levels, aggressive tumor phenotype, poor survival, and cisplatin resistance (59). VEGF also plays a role in tumor progression and exerts immunosuppressive effects in OCCC (60).

HNF-1β expression is characteristic of both endometriosis and OCCC, suggesting early differentiation of endometriosis into clear cell lineage. RNA interference studies show that HNF-1β is essential for OCCC cell survival, with its knockdown inducing apoptosis. HNF-1β promotes glycogen synthesis and aerobic glycolysis, contributing to the metabolic reprogramming that supports chemoresistance (61). Despite its importance, no targeted therapies against HNF-1β have yet been developed, highlighting the need for further investigation (62).

The HGFR signaling pathway is critical for cell growth, survival, and motility through activation of RAS-MAPK, PI3K/Akt/mTOR, and JAK/STAT3 pathways (63, 64). HGFR also functionally interacts with HIF-1α, regulating VEGF expression (65).

OCCC frequently exhibits mismatch repair deficiency (MMRd), resulting in accumulation of genetic abnormalities and microsatellite instability (MSI) (66, 67). MSI arises from mutations or methylation in mismatch repair genes such as MLH1, MSH2, and MSH6 (68). ARID1A mutations contribute to MMRd and MSI by disrupting protein interactions with MSH2 and promoting somatic MLH1 methylation (52, 69, 70). High MSI (MSI-H) occurs in 2–20% of ovarian cancers, predominantly in endometrioid carcinoma (EC) and OCCC (71). Diffuse intratumoral stromal inflammation has been reported as a histologic marker of MMRd in OCCC (72). OCCC tumors with MSI-H are highly immunogenic, exhibiting increased tumor-infiltrating lymphocytes (TILs) and elevated PD-L1 expression, which may predict enhanced responsiveness to immune checkpoint blockade (ICB) therapies (71, 73, 74).

**Figure 1:**
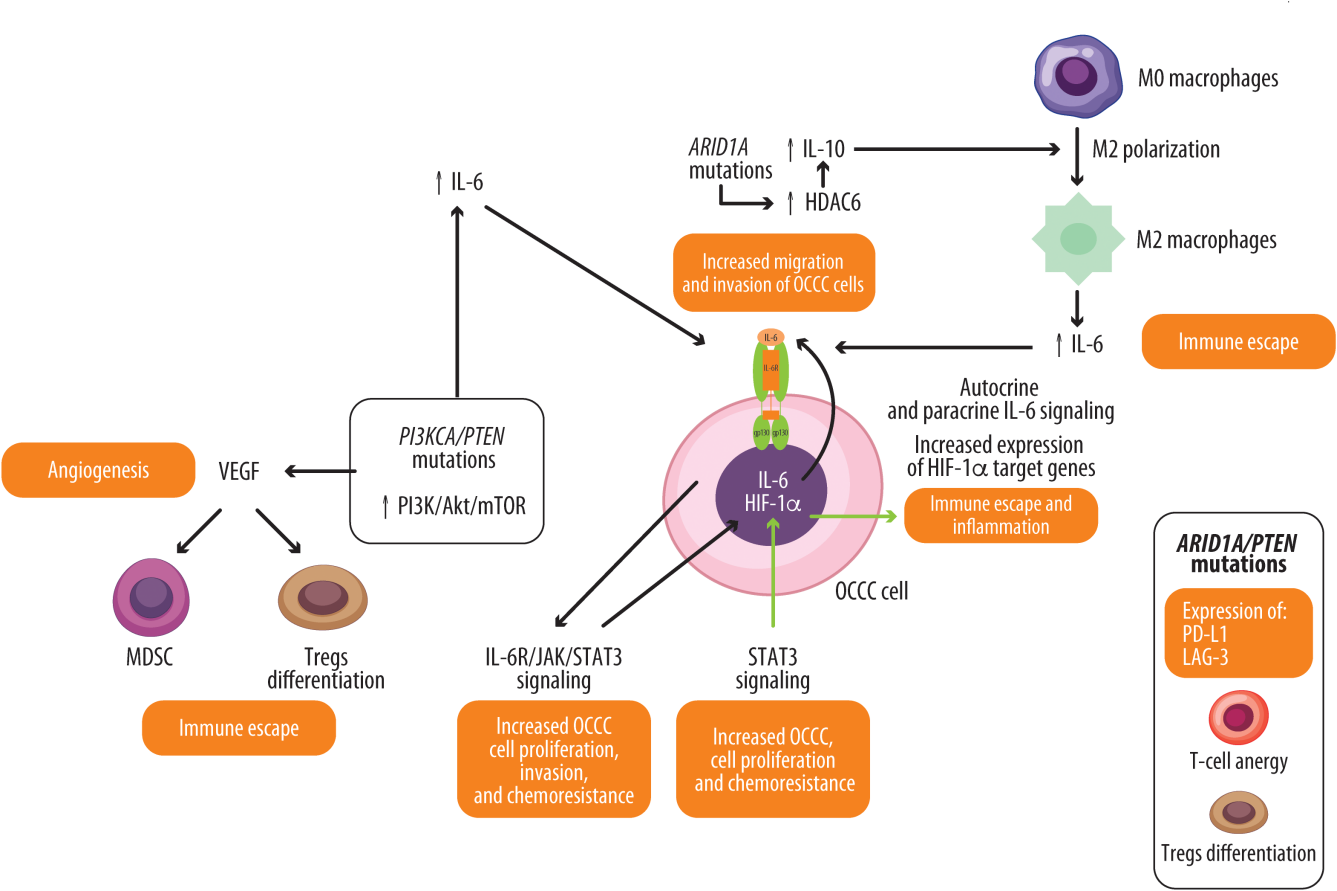
Gene mutations in OCCC and their association with the activation of pro-tumoral signaling pathways. PI3KCA/PTEN mutations induce high levels of PI3K/Akt/mTOR activation, which increases the expression and signaling of IL-6 and the expression of VEGF. VEGF, in turn, promotes angiogenesis and enhances the activity of Tregs and MDSCs, contributing to immunosuppression. IL-6, through its interaction with its receptor, activates STAT3, which enhances the nuclear translocation and transcriptional activity of HIF-1, resulting in chemoresistance and proliferation. Conversely, HIF-1 enhances the activation of STAT3. ARID1A mutations lead to increased levels of HDAC6. HDAC6 increases IL-10 expression, which, in turn, promotes the polarization of macrophages toward an M2 pro-tumoral phenotype, leading to IL-6 secretion and supporting immunosuppression. Concomitant mutations of ARID1A and PTEN promote the expression of immune checkpoints such as LAG-3 and PD-L1, contributing to T-cell anergy and the differentiation of Tregs.

In summary, the molecular landscape of OCCC converges on five major oncogenic pathways: PI3K/Akt/mTOR, HIF-1α/VEGF, HNF-1β, IL-6/STAT3, and HGFR. These interconnected signaling networks orchestrate tumor growth, angiogenesis, immune evasion, metabolic adaptation, and drug resistance, providing a strong rationale for the development of multi-targeted therapeutic strategies (**Figure 1**) (11, 75–77).

### 3.2 The immune tumor microenvironment in OCCC

#### 3.2.1 Anti-tumor immunity and the establishment of an immunosuppressive and pro-tumor TME

The TME constitutes a complex and dynamic milieu comprising cellular components, including cancer cells, endothelial cells, fibroblasts, granulocytes, lymphocytes, and macrophages, embedded in an altered extracellular matrix (ECM) (78). The ECM not only supports tumor architecture but also orchestrates cell-to-cell and cell-to-matrix signaling, and is enriched in inflammatory mediators, chemokines, and matrix-degrading enzymes, such as metalloproteinases, which drive matrix breakdown, neoplastic invasion, and angiogenic processes (79, 80). The immunomodulatory potential of the TME depends on its cellular composition, influencing both innate and adaptive immune responses (81). Among its immune components, TILs are critical for identifying and eradicating malignant cells via both innate defenses and antigen-specific mechanisms (82). Antigen-presenting cells (APCs) initiate adaptive responses by activating CD4^+^ T helper cells (Th1, Th2, Th17), regulatory T cells (Tregs), and cytotoxic T lymphocytes (CTLs) (75, 83, 84). Innate immunity relies on natural killer (NK) cells and macrophages. NK cells eliminate tumors with reduced major histocompatibility complex (MHC) expression (85), while M1-polarized macrophages produce pro-inflammatory cytokines (e.g., IL-6, IL-12, IFN-γ) and facilitate antigen presentation (86). However, the TME often impairs immune responses, recruiting Tregs, myeloid-derived suppressor cells (MDSCs), and tumor-associated macrophages (TAMs), which promote immunosuppression and tumor progression (87–89). Immune evasion is supported by the expression of immune checkpoints, including cytotoxic T-lymphocyte antigen 4 (CTLA-4), programmed cell death protein 1 (PD-1), and lymphocyte activation gene 3 (LAG-3), which inhibit T-cell activation (90–92). Hypoxia, a hallmark of the TME due to abnormal vasculature and high tumor cell proliferation, exacerbates immunosuppression by recruiting MDSCs, TAMs, and Tregs (93). HIFs, particularly HIF-1α, promote tumor cell survival, inflammation, and immune escape (94). Tumor-associated neutrophils and cancer-associated fibroblasts (CAFs) further reshape the TME, enhancing tumor growth, invasion, angiogenesis, and drug resistance (95, 96). In summary, the TME fosters tumor progression and immune evasion by altering immune cell phenotypes, creating hypoxic conditions, and facilitating intercellular communication, ultimately leading to metastatic potential and treatment resistance (97, 98).

#### 3.2.2 Characteristics of the TME in OCCC

As for many types of cancer, the TME plays a crucial role in driving the aggressiveness and immune escape of ovarian cancer (99). Specifically, the TME in OCCC is characterized by a hypoxic environment that supports glycogen synthesis and accumulation (46). Chemoresistant subpopulations of OCCC cells exhibiting elevated HIF activity have been identified in areas enriched with CAFs that display a myofibroblastic phenotype (myCAFs). Indeed, myCAFs enhance OCCC chemoresistance and induce HIF-1α activity, mediated by platelet-derived growth factor (PDGF) signaling through PDGF receptors expressed by CAFs (100).

The OCCC TME displays a high-iron content feature, thought to derive from CD10-negative, endometriosis-derived mesenchymal stem cells (enMSCs) that support tumor growth by donating iron. These enMSCs overexpress iron-export proteins, increasing labile intracellular iron levels, which promote OCCC cell proliferation while shielding them from iron chelation therapies. However, this enhanced iron transfer simultaneously renders OCCC cells vulnerable to ferroptosis, identifying a potential therapeutic target for treatment (101).

Regarding immune escape, several studies have identified aberrant expression of various immune checkpoint genes in OCCC, including CTLA-4, PD-1, PD-L1, LAG-3, and T-cell immunoglobulin and mucin domain-containing protein 3 (TIM-3), positioning these as potential targets for ICB therapies. Collectively, these findings suggest that OCCC may be particularly responsive to ICB strategies (102). PD-L1 have been shown to be expressed in ovarian tumors and to represent negative prognostic markers. Moreover, PD-L1 promote immune evasion by inducing T-cell anergy or apoptosis, favoring tumor immune escape (103). In OCCC, PD-L1 expression is driven by *ARID1A* mutations (104, 105) and the hyperactivation of the PI3K/Akt pathway (51). The interaction of PD-L1 with PD-1 on T cells triggers inhibitory signals that suppress activated T lymphocytes, promote T-cell anergy and apoptosis, and ultimately lead to the activation and expansion of Tregs (106). VEGF expression plays an important role in the tumor progression of OCCC and, in addition to promoting angiogenesis, acts as an immunosuppressive agent by impairing T-cell function and APC function, and activating Tregs and MDSCs (60). The increased presence of Tregs within the OCCC TME has been associated with tumor progression and resistance across multiple stages of the disease (107). Moreover, frequent alteration of the PI3K/Akt/mTOR pathway observed in OCCC not only upregulates PD-L1 expression but also enhances the expression of LAG-3. This dual activation promotes resistance to cytotoxic T-cell-induced apoptosis, enhances Treg function, and enables evasion from death receptor-mediated signaling (108–111).

The expression of LAG-3 in TILs has been reported in OCCC and is associated with a poor prognosis (112). Furthermore, recent studies showed that the *ARID1A*^6488delG^ mutation (113) induces M2 polarization of macrophages through IL-10, thus contributing to differentiation into TAMs and immunosuppressive conditions (40).

**Figure 2:**
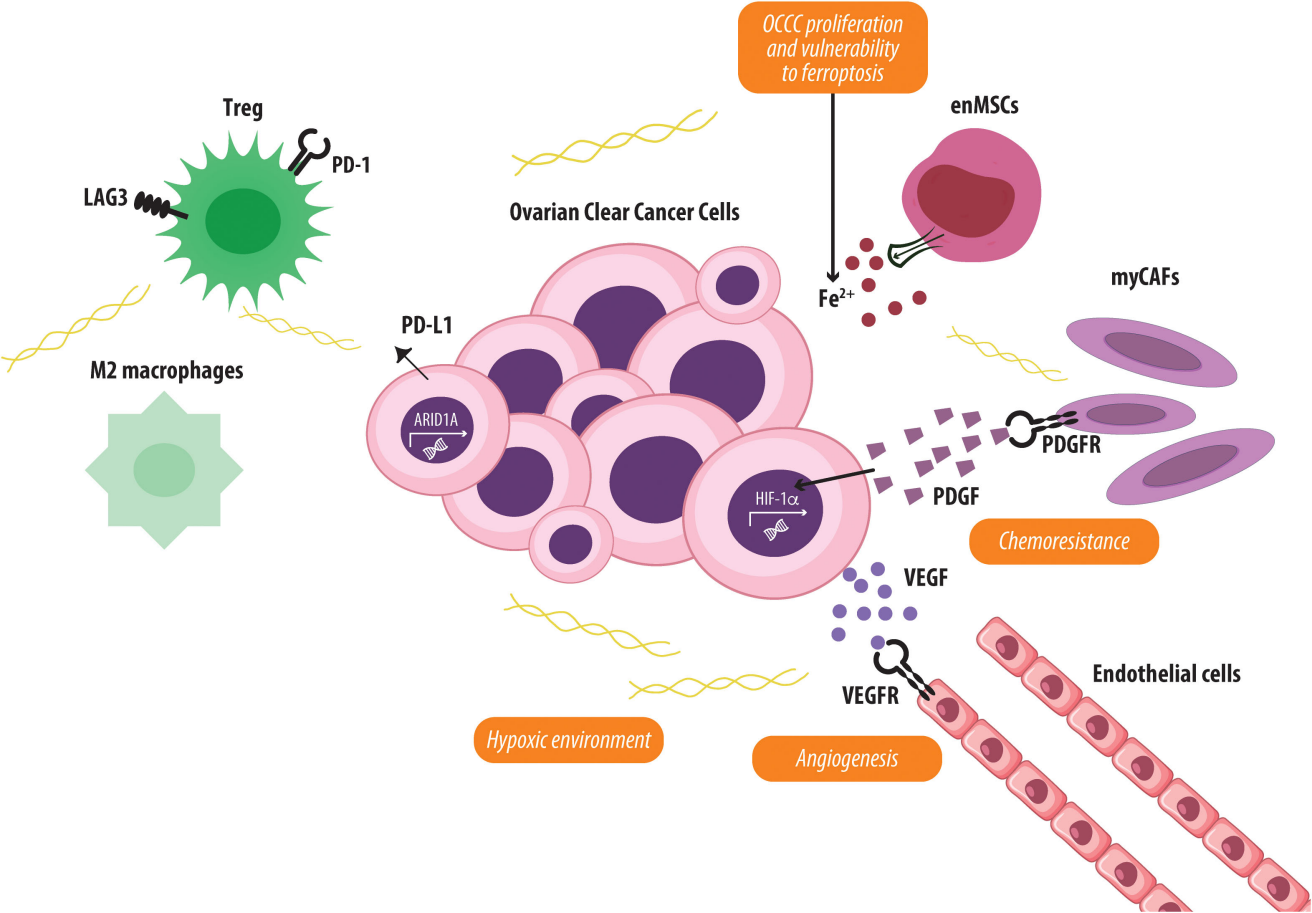
Characteristics of the OCCC TME. The OCCC TME is highly immunosuppressive and pro-tumorigenic. This is characterized by hypoxia and angiogenesis driven by VEGF and HIF-1α, which promote tumor growth and chemoresistance. Endometriosis-derived mesenchymal stem cells (enMSCs) enrich the microenvironment with iron, sustaining OCCC proliferation but also conferring vulnerability to ferroptosis. Cancer-associated fibroblasts with a myofibroblastic phenotype (myCAFs) contribute to chemoresistance and enhance HIF-1α activity through PDGF signaling. The immune landscape includes regulatory T cells (Tregs) and M2-polarized macrophages, which suppress anti-tumor responses, alongside aberrant expression of immune checkpoints such as PD-1, PD-L1, and LAG-3 that promote T-cell anergy. Together, these features foster immune escape, therapeutic resistance, and disease progression.

In summary, the OCCC TME is characterized by a hypoxic, iron-rich, and immunosuppressive profile, featuring the infiltration of Tregs and TAMs (**Figure 2**) (103, 114, 115).

### 3.3 Immune therapeutic strategies against OCCC

Despite the strong rationale for response to immunotherapy in OCCC, its clinical efficacy in EOC remains limited. Nevertheless, several ongoing clinical trials are exploring the use of immune checkpoint inhibitors (ICIs) in patients with advanced OCCC. Additionally, anti-angiogenic therapies have demonstrated potential in enhancing the efficacy of immunotherapy by modifying the TME and directly influencing immune effector cells (116).

In this regard, in newly diagnosed stage III or IV ovarian carcinoma, the IMagyn050/GOG 3015/ENGOT-OV39 phase III trial (NCT03038100) evaluated the addition of the PD-L1 inhibitor atezolizumab to standard platinum-based chemotherapy and bevacizumab. While no significant improvements were seen in PFS or OS (co-primary endpoints), *post hoc* subgroup analyses reported a numerical increase in PFS with the addition of atezolizumab in non-high grade serous histology, which included OCCC (117). The phase III study, NINJA (JapicCTI-153004), compared nivolumab (PD-1 inhibitor) with chemotherapy (gemcitabine or pegylated liposomal doxorubicin) in patients with platinum-resistant ovarian cancer. Although nivolumab was better tolerated and associated with a longer duration of response, it did not improve OS and showed inferior PFS compared with chemotherapy (118).

A phase II trial evaluated pembrolizumab in two cohorts of patients with recurrent ovarian cancer, stratified by treatment history and platinum-free intervals (NCT02674061). The overall response rate (ORR) was low—7.4% in cohort A (1–3 prior therapies) and 9.9% in cohort B (4–6 prior therapies)—with modest disease control rates (approximately 37%) in both groups. PD-L1 expression was associated with slightly higher responses (ORR 10%). PFS was 2.1 months, and OS was 17.6 months in cohort B (not reached in cohort A) (119).

The combination of nivolumab and ipilimumab (CTLA-4 inhibitor) in EOC yielded a higher ORR and slightly prolonged PFS than nivolumab alone, with manageable toxicity (NCT02498600). Notably, patients with OCCC demonstrated a fivefold higher ORR than other histologic subtypes, although the sample size was small (12% OCCC) (120).

Further supporting this trend, the results of the BrUOG 354 trial were absolutely promising and surprising. The BrUOG 354 trial (NCT03355976), a randomized two-stage, phase II study, evaluated nivolumab monotherapy versus nivolumab in combination with ipilimumab (nivolumab/ipilimumab) in patients with relapsed extra-renal CCC, including ovarian, endometrial, and cervical primaries. All participants had gynecologic tumors, with 36 (82%) diagnosed with OCCC. Patients had received a median of one prior line of therapy (range 1-7). The ORR was 14.3% (two partial responses) with nivolumab and 33% (four complete and six partial responses) with nivolumab/ipilimumab. Median PFS was 2.2 months (95% CI: 1.2–3.4) with nivolumab and 5.6 months (95% CI: 1.6–29.1) with nivolumab/ipilimumab. Median OS was 17.0 months (95% CI: 2.1–NR) with nivolumab and 24.6 months (95% CI: 5.9–NR) with nivolumab/ipilimumab. Grade 3 treatment-related adverse events occurred in 21% of patients on nivolumab and 47% of those on nivolumab/ipilimumab (including two grade 4 pancreatic enzyme elevations), with no treatment-related deaths reported. These results highlight the meaningful and durable clinical activity of nivolumab/ipilimumab, particularly in OCCC, and support its further evaluation in this historically chemotherapy-resistant population (121).

There are several ongoing clinical trials of anti-angiogenic therapy in combination with ICIs recruiting patients with OCCC. The combination of pembrolizumab (PD-1 inhibitor) with lenvatinib, an oral multikinase inhibitor that targets VEGF receptor, fibroblast growth factor receptor 1–3, PDGF receptor, RET, and KIT, has been evaluated in several gynecologic malignancies and is currently approved by the US Food and Drug Administration (FDA) for use in advanced, microsatellite stable, MMR proficient endometrial carcinoma (122). This combination has been shown to improve PFS and OS in endometrial cancer regardless of histologic subtypes. However, a *post-hoc* analysis suggests a specific clinical benefit in the clear cell histological subtype (123). In this regard, two phase II clinical trials on the combination of lenvatinib plus pembrolizumab are currently recruiting patients with recurrent or persistent OCCC who have received at least one prior line of platinum-based chemotherapy. The LARA phase II trial (Singapore and South Korea, NCT04699071) evaluates the combination of pembrolizumab and lenvatinib in recurrent clear cell gynecological cancer. The preliminary efficacy reported achieving an objective response in four out of 15 patients in the first 24 weeks (ORR at 24 weeks, 26.7%; 95% CI: 7.8–55.1). The median PFS was 12 weeks (95% CI: 5.4–24.4); PFS at 12 and 24 weeks was achieved in 46.7% (95% CI: 21.2–68.7) and 33.3% (95% CI: 12.2–56.4) of patients, respectively (124).

Similarly, the NCT05296512 trial is recruiting patients with recurrent or persistent OCCC in the USA to undergo an experimental combination of pembrolizumab and lenvatinib (125, 126). Interestingly, McNamara and colleagues reported the case of a patient with recurrent treatment-resistant OCCC with *ARID1A/PIK3CA* mutations after failing standard and experimental treatments, who had a partial and durable response with pembrolizumab and lenvatinib over 7 months of treatment approved on a compassionate basis (127). Regarding the use of immunotherapy, two recent single-arm, multicenter, phase II trials reported interesting data at the European Society for Medical Oncology (ESMO) 2022 congress. The British PEACOCC (NCT03425565) study enrolled 49 recurrent CCC, of whom 85.4% were OCCC. The study exhibited the promising efficacy of pembrolizumab monotherapy with a 12-week PFS rate of 43.8% (95% CI: 31.5–56.6) (128).

Furthermore, the Chinese INOVA (NCT04735861) study investigated the potential benefit of combining sintilimab (PD-1 inhibitor) and bevacizumab for recurrent or persistent OCCC (129). Preliminary results on 23 patients (of whom 18 were platinum-resistant and 20 with radiological evaluation) reported an ORR of 40% (one complete and seven partial responses; 95% CI: 19.1–63.9) and a disease control rate of 75% (eight partial responses, seven stable diseases; 95% CI: 50.9–91.3%) (130).

Notably, MITO 27 (NCT04375956), a prospective non-randomized phase II study, evaluates the use of pembrolizumab only for patients with a combined positive score >1 in recurrent, platinum-resistant OC, including clear cell histology (131).

The MOCCA trial (NCT03405454) testing durvalumab, an anti-PD-L1 monoclonal antibody, versus standard chemotherapy in patients with recurrent OCCC showed no significant difference in PFS, ORR, or clinical benefit rate. However, correlative translational analyses to elucidate potential predictive biomarkers of response and resistance are ongoing (132).

Finally, BOUQUET (NCT04931342) is a phase II, open-label, non-randomized, multicenter, platform study evaluating biomarker-driven treatments in patients with persistent or recurrent ovarian, fallopian tube, or primary peritoneal tumors of rare epithelial histology, including OCCC (133). The treatment arm to which eligible patients are assigned will be determined by the biomarker profile of their tumor. Recently, the first interim results have been reported from the cobimetinib arm, a mitogen-activated extracellular signal-regulated kinase 1 (MEK1) inhibitor and from the role of combination of atezolizumab (anti-PD-L1) plus bevacizumab (anti-VEGF) arm. Specifically, as of the clinical cut-off date, five patients with OCCC had received cobimetinib, and three patients with OCCC had received the combination with bevacizumab and atezolizumab. All patients were heavily pretreated. Confirmed objective response rates were 16% with cobimetinib and 14% with atezolizumab plus bevacizumab. This trial continues to evaluate biomarker-driven therapies for rare epithelial ovarian cancer (134).

Recently, a press release from Merck announced that the phase 3 KEYNOTE-B96/ENGOT-ov65 trial (NCT05116189) assessing pembrolizumab plus paclitaxel with or without bevacizumab reached the primary endpoint of PFS among patients with platinum-resistant ovarian cancer across the all-comer and PD-L1-positive populations.

**Table 1** summarizes the findings of the clinical trials presented.

**Table 1:** Clinical trials with immune checkpoint inhibitors enrolling patients with advanced OCCC.

Clinical trial	Drug	Mechanism of action	Phase	Condition or disease	Drug combinations	Recruitment status
NCT03405454 (MOCCA) (132)	Durvalumab	Anti-PD-L1 mAb	II	OCCC	Durvalumab	Unknown
NCT03355976 (BrUOG 354) (135)	Nivolumab Ipilimumab	Anti-PD-1 mAb Anti-CTLA-4 mAb	II	Ovarian and extra-renal clear cell carcinomas	Nivolumab ± ipilimumab	Active, not recruiting
NCT04699071 (LARA) (136)	Pembrolizumab	Anti-PD-1 mAb	II	Recurrent gynecological clear cell carcinoma	Pembrolizumab + lenvatinib	Unknown (Singapore)
NCT05296512 (122)	Pembrolizumab	Anti-PD-1 mAb	II	OCCC	Pembrolizumab + lenvatinib	Recruiting (USA)
NCT05026606 (EON) (137)	Nivolumab Etigilimab	Anti-PD-1 mAb Anti-TIGIT mAb	II	OCCC	Nivolumab + Etigilimab	Recruiting
NCT05032040 (138)	XmAb20717 (Vudalimab)	Bispecific antibody targeting PD-1 and CTLA-4	II	Refractory clear cell ovarian, endometrial, or peritoneal cancer	Vudalimab	Recruiting
NCT03425565 (PEACOCC) (128)	Pembrolizumab	Anti-PD-1 mAb	II	Recurrent gynecological clear cell carcinoma	Pembrolizumab	Active, not recruiting
NCT04735861 (INOVA) (129)	Sintilimab Bevacizumab	Anti-PD-1 mAb Anti-VEGF mAb	II	OCCC	Sintilimab + bevacizumab	Completed (China)
NCT04931342 (BOUQUET) (133)	Atezolizumab Bevacizumab	Anti-PD-L1 mAb Anti-VEGF mAb	II	Rare epithelial ovarian tumors (OCCC)	Atezolizumab + bevacizumab	Active, not recruiting
NCT04375956 (MITO 27) (131)	Pembrolizumab	Anti-PD-1 mAb	II	Recurrent, platinum-resistant, CPS >1 positive ovarian cancers (OCCC)	Pembrolizumab	Recruiting

NCT, National Clinical Trials identifier; OCCC, ovarian clear cell carcinoma; mAb, monoclonal antibody; PD-L1, programmed death-ligand 1; PD-1, programmed cell death protein 1; CTLA-4, cytotoxic T-lymphocyte antigen 4; TIGIT, T-cell immunoreceptor with Ig and ITIM domains; VEGF, vascular endothelial growth factor; CPS, combined positive score.

### 3.4 Possible new therapeutic strategies and future perspectives in OCCC treatment

**Figure 3:**
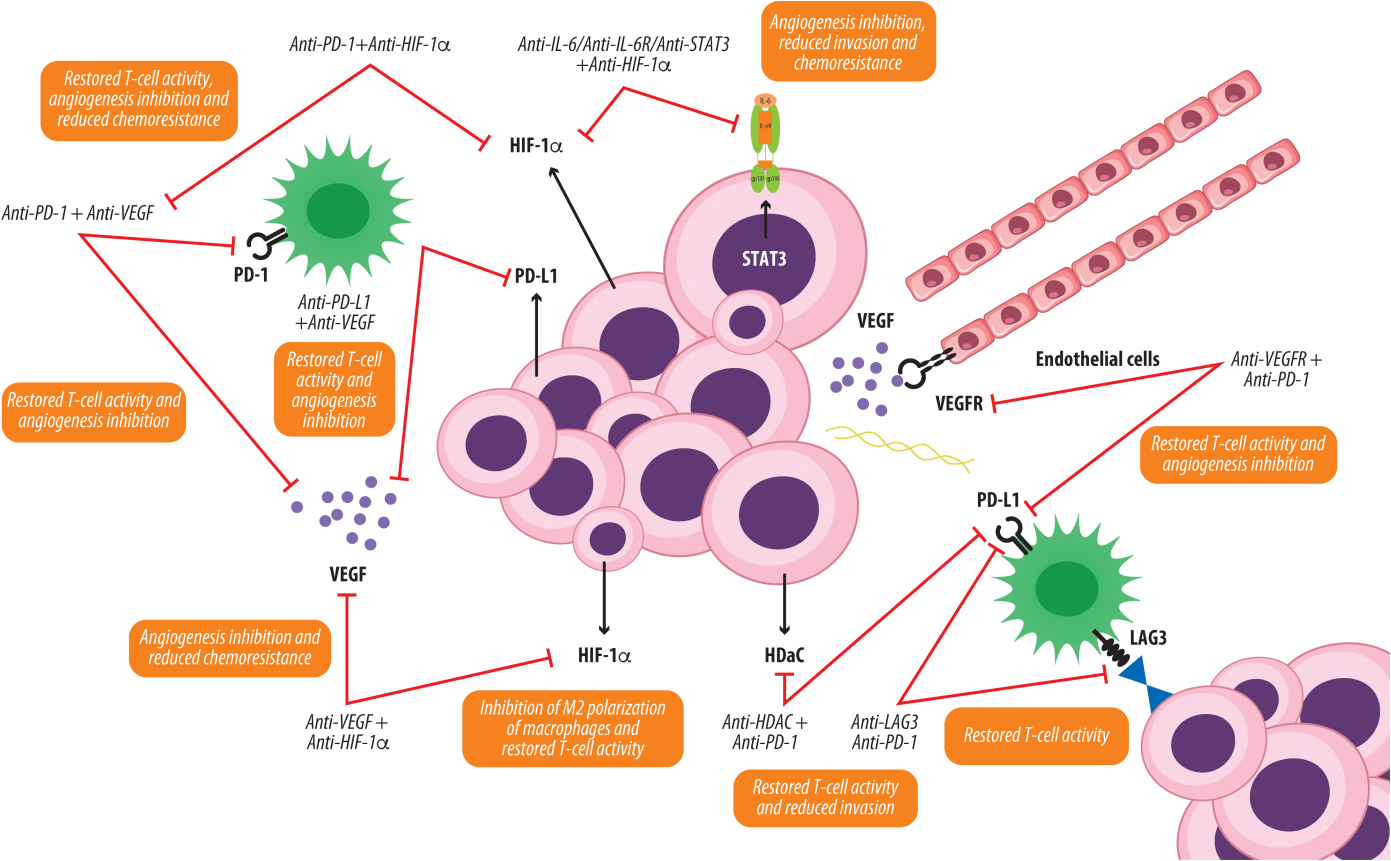
Potential therapeutic strategies targeting the OCCC TME. The diagram illustrates the multifaceted effects of combining targeted therapies counteracting key tumor-promoting pathways (IL-6/IL-6R/STAT3, HIF-1α, VEGF/VEGFR, PD-1/PD-L1, LAG3, and HDAC) on tumor growth, immune modulation, angiogenesis, and chemoresistance. Checkpoint inhibitors (anti-PD-1, anti-PD-L1, and anti-LAG-3) restore T-cell activation and effector function, while anti-VEGF and anti-VEGFR therapies inhibit angiogenesis and tumor vascularization. Inhibition of the IL-6 signaling pathway with anti-IL-6, anti-IL-6R, or anti-STAT3 antibodies, alone or in combination with anti-HIF-1α, leads to reduced angiogenesis, invasion, and chemoresistance. Additional strategies such as anti-HDAC agents might further enhance T-cell activity and reduce immunosuppression macrophage polarization. The combined effects of these therapeutic approaches potentially result in a more immunostimulatory tumor microenvironment and attenuation of OCCC progression.

Given the complexity of the OCCC TME, therapeutic strategies that combine ICIs with agents targeting key molecules implicated in tumor aggressiveness such as VEGF, HIF-1α, IL-6, IL-10, PI3K, and HDAC6 show considerable promise (**Figure 3**).

Notably, an *in vivo* study employing animal models demonstrated that combining HDAC6 inhibition via ACY1215 with ICIs constitutes a potential therapeutic approach for ARID1A-mutated OCCC, effectively limiting tumor progression through a cytotoxic T-cell-dependent mechanism (139).

EZN-2208, an inhibitor of HIF-1α, has displayed anti-tumor activity both as monotherapy and in combination with bevacizumab in phase I clinical trials involving refractory solid tumors (140, 141). Additionally, an *in vitro* study revealed that PX-478, another HIF-1α inhibitor, enhanced T cell-mediated tumor cell killing when combined with ICIs in non-small cell lung cancer models (142). Beyond ICIs, HIF-1α inhibitors may also be combined with anti-angiogenic agents, representing a promising strategy in OCCC. Considering the interplay between HIF-1α and IL-6, dual inhibition targeting IL-6, its receptor, or downstream STAT3 signaling alongside HIF-1α may provide an effective therapeutic avenue.

Co-inhibition of PD-1 and CTLA-4 has demonstrated antitumor efficacy in OCCC patients; however, this approach is often limited by severe immune-related adverse events (120, 143). Given these limitations, a randomized phase III trial in melanoma showed that combined inhibition of PD-1 and LAG-3 resulted in improved clinical outcomes (78), suggesting that targeting PD-1 and LAG-3, rather than PD-1 and CTLA-4, could be a safer and potentially more effective strategy in OCCC. Currently, only a few early-phase clinical studies (phases 1 and 2) have investigated anti-LAG-3 monoclonal antibodies (mAbs) as monotherapy or in combination with other ICIs such as anti-PD-1 and anti-CTLA-4 in advanced solid tumors, including ovarian cancer. Despite strong scientific rationale, no clinical trials specifically evaluating anti-LAG-3 therapy in OCCC have been reported to date.

Moreover, inhibition of IL-6 signaling through the IL-6 receptor alpha (IL-6Rα) inhibitor tocilizumab has been shown to enhance the efficacy of cytotoxic chemotherapy by promoting cytotoxic T lymphocyte (CTL)-mediated antitumor responses, concurrently downregulating PD-L1 expression and potentially augmenting responses to ICIs (144).

IL-10 blockade—achieved using soluble IL-10 receptors (145), peptide-based IL-10 receptor antagonists (146), or oligonucleotide-based inhibitors (147)—may further potentiate antitumor immunity in OCCC by augmenting T-cell responses and reprogramming tumor-associated macrophages (TAMs). These immunomodulatory effects could be synergistically enhanced when combined with IL-6 and VEGF inhibition or ICIs.

Inhibition of the PI3K/Akt/mTOR and ERK1/2 signaling pathways has been shown to reduce progranulin (PGRN) expression in ovarian cancer cells, suggesting a potential strategy to overcome chemoresistance in OCCC. Alpelisib (BYL719), the first oral isoform-selective PI3K inhibitor targeting the p110α isoform of wild-type PI3Kα, has received FDA and EMA approval for metastatic breast cancer treatment (148). Alpelisib has also been employed in advanced gynecologic malignancies harboring PIK3CA mutations, including ovarian cancer. While the most notable clinical benefit has been observed in endometrial cancer patients (149), including a documented case of *PIK3CA*-mutated endometrial cancer achieving a clinically meaningful response (150), in ovarian cancer cohorts, patients with OCCC demonstrated a disease control rate of 50% (2 stable disease, 1 partial response) (149).

Therefore, it is crucial that translational research focuses on finding valid predictive biomarkers of response to new personalized therapies in addition to the potential expression of PD-L1 and the critical importance of designing clinical trials specifically dedicated to the OCCC histotype.

## 4 Conclusion

OCCC represents a challenging subtype of EOC, owing to its distinct molecular features, limited response to conventional therapies, a poor prognosis and highly immunosuppressive TME. Frequent genetic and molecular alterations in OCCC, including *ARID1A* and *PIK3CA* mutations, lead to hyperactivation of the PI3K/Akt/mTOR pathway and the overexpression of IL-6, IL-10, HDAC6, VEGF, and HIF-1α, collectively driving its aggressive behavior, chemoresistance, and immune evasion.

The immunosuppressive TME of OCCC, characterized by a high infiltration of Tregs, TAMs, and elevated levels of immune checkpoint molecules such as PD-L1 and LAG-3, could be an attractive target for the immune therapeutic intervention.

These features underline the complexity of managing OCCC and highlight the urgent need for innovative therapeutic approaches, including immunotherapy.

Despite increasing understanding of OCCC pathophysiology, current therapeutic strategies, including ICIs and anti-angiogenic agents, have shown limited clinical efficacy in this cancer type. Nevertheless, emerging evidence from ongoing clinical trials suggests potential benefits from combinatorial therapies targeting multiple pathways. For instance, the combination of pembrolizumab with lenvatinib or VEGF inhibitors has demonstrated promising activity in subsets of OCCC patients, underscoring the importance of multi-targeted approaches. Similarly, preclinical studies suggest that inhibiting key molecules such as IL-6, HIF-1α, HDAC6, or IL-10, in combination with ICIs, could overcome resistance mechanisms and enhance treatment efficacy. Future therapeutic strategies should focus on leveraging the intricate interplay between the genetic and immunological features of OCCC. Targeting *ARID1A*-mutated tumors through specific inhibitors of HDAC6 or IL-6 signaling could reprogram the immunosuppressive TME and restore T-cell-mediated cytotoxicity. Additionally, continued exploration of innovative combinations, such as dual ICB (e.g., PD-1 and LAG-3 inhibition) may further expand the therapeutic armamentarium for this malignancy.

In conclusion, while OCCC remains a formidable clinical challenge, advances in molecular and immunologic research offer hope for more effective and durable therapeutic options. Future studies should prioritize the integration of targeted therapies with immunomodulatory agents to address the multifaceted nature of OCCC, ultimately improving outcomes for patients with this rare and aggressive cancer.
